# Real-World clinical outcomes of asthma patients switched from reslizumab to mepolizumab or benralizumab

**DOI:** 10.3389/falgy.2022.1052339

**Published:** 2023-01-04

**Authors:** Laura J. Walsh, Deborah Casey, Punitha Vairamani, Fiona Arnott, Barry J. Plant, Desmond M. Murphy

**Affiliations:** ^1^ Department of Respiratory Medicine, Cork University Hospital, Cork, Ireland; ^2^ Clinical Research Facility, University College Cork, Cork, Ireland

**Keywords:** asthma, biological therapy, exacerbation, ACQ, real-word

## Abstract

**Introduction:**

Approximately 3%–10% of asthma patients will remain uncontrolled despite maximum, optimal conventional therapy. Treatment of severe refractory asthma often involves the use of targeted biological therapy. Randomised controlled trials have shown improvements in clinical parameters with these treatments but real-world data is lacking.

**Methods:**

The clinical parameters, frequency of exacerbations, number of hospital admissions, asthma control questionnaire score (ACQ), forced expiratory volume in one second (FEV_1_) and maintenance oral corticosteroid (OCS) dose of twenty asthma patients switched from reslizumab to benralizumab or mepolizumab at 1 year prior and 6 months after switching were compared, with adjustments for time.

**Results:**

The mean frequency of exacerbations (0.35 v 0.3) and the mean ACQ were essentially unchanged (1.6 v 1.5) following the switch. The number of hospital admissions was one in the 6 months post switch compared to one in 1-year pre switch. 25% of patients were on maintenance OCS before and after switching but one patient required an increased dose post switch resulting in an increase in the mean maintenance OCS dose (1.6 mg to 2.4 mg). The mean FEV_1_ was unchanged (80% v 77.9%) six months post switching. Regarding asthma control (*n* = 19), 47.4% were controlled pre and post switch (ACQ < 1.5), 36.8% remained uncontrolled despite switching, 10.5% improved control while 5.3% disimproved.

**Conclusion:**

We present real-world clinical outcomes of asthma patients switched from reslizumab to either benralizumab or mepolizumab without a loss of clinical effectiveness in the majority.

## Introduction

Asthma is a common, chronic, heterogeneous disease characterised by airways inflammation, bronchial hyperresponsiveness and airflow reversibility (1). Ireland has the fourth highest prevalence of asthma worldwide, with over 470,000 people diagnosed with asthma (2). Asthma can be subdivided into different endotypes based on its pathophysiology including an inflammatory or Type-2 (T2) endotype as well as a non T2 endotype (3). The T2 endotype is so called because of the role type-2 T helper cells have in activating an inflammatory cascade which releases pro-inflammatory mediators such as interleukin-5 (IL-5), IL-4 and IL-13 (3). Innate lymphoid cells (ILC's) are another source of proinflammatory cells and are also activated in T2 asthma (3).

Approximately 3%–10% of asthma patients will remain uncontrolled despite maximum, optimal conventional therapy (4). This group, known as severe refractory asthmatics, have derived clinical benefit from drugs aimed at the interleukins and receptors activated in T2 inflammatory pathways. Current available treatments for severe refractory asthmatics include monoclonal antibodies (mAb) which target IL-5 (reslizumab and mepolizumab), the IL-5 receptor (benralizumab), the IL-4/13 receptor (dupilumab) or immunoglobulin E (IgE) (omalizumab) (5).

Reslizumab is an IgG subclass 4 *κ* mAb, given intravenously, which targets IL-5 in patients with severe eosinophilic asthma (SEA) (6). It has been shown in both clinical trials and real-world studies to reduce exacerbation frequency and oral corticosteroid (OCS) use in severe asthmatics (7–9).

Mepolizumab is a mAb which blocks the interaction between the *α*-subunit and the IL-5 receptor on the surface of the eosinophil thereby preventing eosinophil maturation and activation (5, 10). Clinical trials demonstrated mepolizumab's ability to reduce the frequency of exacerbation and reduce OCS use in those with SEA (11–13). These results were also observed in numerous real-world studies of mepolizumab (14–17) and indeed extension studies of the original trails confirmed mepolizumab's favourable long-term safety profile (18–20). Furthermore, a small real-world study has also highlighted mepolizumab's potential use in severe asthmatic patients with co-existing bronchiectasis (21). Benralizumab targets the IL-5 receptor so therefore has a different mode of action to the previous mAb's. Trials such as CALIMA and SIROCCO have demonstrated that treatment with benralizumab can result in a reduction of exacerbation frequency, improvements in the forced expiratory volume in one second (FEV_1_) and a reduction in the use of OCS in severe asthma patients, with similar results in real world studies (22–27).

Our institute has previously reported on the real-world clinical outcomes of ten patients who switched from omalizumab to anti-IL-5 therapy (*n* = 6 benralizumab and *n* = 4 mepolizumab) due to sub-optimal control on omalizumab with significant reductions in community exacerbation rate and FEV_1_ and non-significant improvements in OCS use one year post switching to anti-IL-5 therapy (28). This study was in line with other studies which assessed a switch from omalizumab to mepolizumab in patients with SEA who were not adequately controlled on omalizumab with a reduction in exacerbation frequency and OCS use and improvements in FEV_1_ and asthma control scores (29).

Switching between agents occurs for a number of reasons including clinical ineffectiveness, patient choice and ease of administration. While some patients do well post switch and improve their asthma control, real world data has also highlighted that patients can also partially respond or deteriorate following a change in their biological therapy (30).

Studies have identified various factors which influence an agent's effectiveness with some showing that the need for daily OCS, adult-onset asthma or shorter asthma duration and nasal polyposis seem to confer a poorer response to biologic therapy (30) while the presence of nasal polyposis is predictive of a favorable response in other studies (31, 32). Real world data is therefore very valuable and aids a clinician to make a more informed choice when commencing or switching patients on biological treatment.

Here we present real-world clinical outcomes of twenty patients with SEA who were previously established on reslizumab for at least a year and then switched to either benralizumab or mepolizumab. Real world data regarding switching from reslizumab is not widely available in the literature, therefore, we hope that this current report will be clinically useful.

## Methods

A retrospective, observational, single centre review of the clinical outcomes of twenty patients aged ≥18 years old who attend the difficult to control asthma clinic at Cork University Hospital, Ireland, a regional, academic, tertiary referral centre was carried out. All patients had a diagnosis of severe eosinophilic asthma (SEA) and met the criteria required to be prescribed the relevant biological agent. All patients had been established on reslizumab for at least 1 year prior to switching to either mepolizumab or benralizumab between October 2021 and February 2022. The indication for switching was for easier administration as both mepolizumab and benralizumab can be self-administered subcutaneously whereas reslizumab requires day case admission to hospital for intravenous administration. The aim of this review was to establish if clinical effectiveness was maintained after switching biological therapy. Patients had commenced reslizumab as an initial therapy as it was the first to be available to our institute through an early access programme.

An exacerbation was defined as a need for steroids and/or antibiotics or a doubling of maintenance steroids. Patient characteristics such as the number of exacerbations and the number of hospital admissions experienced by the patient 1 year prior to switching and 6 months after switching were recorded. Results were adjusted for the different time periods. An asthma control questionnaire score (ACQ-7) (33) and FEV_1_ were recorded 1 year prior to switching and then again 6 months post switching and compared. Maintenance OCS use was also recorded at 1-year pre and 6 months post switching. Patient's co-morbidities and other markers of atopy such as IgE and fractional concentration of exhaled nitric oxide (FeNO) were not recorded as part of this study. All patients continued to attend the asthma clinic for surveillance.

Ethical approval for this study was granted by the Cork Research Ethics Committee, Cork, Ireland.

### Statistical analysis

Statistical analysis was performed using GraphPad Prism software 9.1.0 (La Jolla, California, USA). Descriptive statistics were used to analyse patient characteristics. Normally distributed continuous data was described using means, standard deviations (SD), medians and interquartile ranges (IQR). The categorical variables were reported as number of events and frequency. Patient characteristics were compared using independent sample t tests for continuous variables. A *p* value of ≤ 0.05 was considered significant.

## Results

Twenty patients were included in this analysis with a mean age of 61.5 years. 75% of the cohort were female. The average time patients were treated with reslizumab before switching to an alternative was 47.1 months (Range 17–56 months). 25% of patients required maintenance OCS at baseline both before and after the switch with one patient requiring an increased dose of OCS post switch while all others were maintained at an unchanged dose. Table 1 shows the results of the five clinical parameters recorded in this study pre and post switching from reslizumab to either benralizumab or mepolizumab, with adjustments made for frequency of exacerbation and hospital admission due to a difference in time periods. The number of hospital admissions recorded was one in the year prior to switching (0.5 adjusted) and one in the six months post switching, although in different patients. The number of exacerbations remained the same (0.35 v 0.3, *p* = 0.78). Maintenance OCS dose increased but this was due to one patient requiring an increase in dosing from 15 mg to 25 mg during the study period. No patient reduced or completely removed the need for OCS as a result of switching biological therapy.

**Table 1:** Comparing the mean values of the five clinical parameters measured in this study 1 year before switching from reslizumab to either benralizumab or mepolizumab to the mean values of the same parameters 6 months after switching.

Clinical Parameter	1-year pre switch, Mean (SD)	6 months post switch, Mean (SD)	*p*-value
Hospital admissions (adjusted)	0.03 (0.11)	0.05 (0.2)	0.67
Exacerbations (adjusted)	0.35 (0.52)	0.3 (0.6)	0.78
Maintenance OCS dose (mg)	1.63 (3.91)	2.37 (6.3)	0.33
FEV_1_ (%) *n* = 19	80 (25.3)	77.89 (28.1)	0.96
ACQ score, *n* = 19	1.6 (1.3)	1.5 (1.5)	0.66

An ACQ < 1.5 indicates good control of asthma symptoms. The ACQ score of one patient at the required time period was missing, therefore results are based on nineteen patients. Regarding asthma control, based on ACQ score 47.4% were controlled pre and post switch, 36.8% remained uncontrolled despite switching, 10.5% improved control while 5.3% disimproved. Next, we looked at improvements in exacerbation frequency which was defined as reduction of exacerbations by at least 50% following the switch to either mepolizumab or benralizumab, adjusted for time. Here we found that 50% had no exacerbations pre and post switch, 10% were unchanged, 25% reduced their exacerbation frequency following the switch while 15% had an increase in their asthma exacerbations at 6 months post switching from reslizumab.

Sixty percent of patients switched to benralizumab (*n* = 12) while the remaining 40% changed to mepolizumab. Next, we compared the clinical parameters before and after switching specifically for each monoclonal drug. Nine females and three males were switched to benralizumab, with a mean age of 62.4 years while six females and two males switched to mepolizumab with a mean age of 60 years. Table 2 shows the clinical parameters measured for those switched to benralizumab at 1 year before and 6 months after switching and Table 3 shows the same results for those switched to mepolizumab. The data for hospital admissions and frequency of exacerbations is again adjusted for the different time periods observed. For those who switched to benralizumab it is worth noting that there was one hospitalisation post switch and the patient who required extra steroids was also included in this group. For the mepolizumab group there was a reduction in exacerbation frequency from 0.44 to 0.13 (*p* = 0.24) while OCS maintenance dose and ACQ score remained unchanged pre and post switch. There was however a significant reduction in FEV_1_ from 78.8% to 67.6% (*p* = 0.02) post switching.

**Table 2:** Comparing the mean values of the five clinical parameters measured in this study 1 year before switching from reslizumab to benralizumab to the mean values of the same parameters 6 months after switching.

Clinical Parameter Benralizumab (*n* = 12)	1-year pre switch, Mean (SD)	6 months post switch, Mean (SD)	*p*-value
Hospital admissions	0	0.08 (0.3)	0.34
Exacerbations (adjusted)	0.3 (0.45	0.4 (0.6)	0.6
Maintenance OCS dose (mg)	1.04 (2.9)	2.29 (7.2)	0.34
FEV_1_ (%)	80.7 (25.7)	83.9 (29.9)	0.39
ACQ score, *n* = 11	1.6 (1.2)	1.5 (1.5)	0.80

**Table 3:** Comparing the mean values of the five clinical parameters measured in this study 1 year before switching from reslizumab to mepolizumab to the mean values of the same parameters 6 months after switching.

Clinical Parameter Mepolizumab *n* = 8	1-year pre switch, Mean (SD)	6 months post switch, Mean (SD)	*p*-value
Hospital admissions(adjusted)	0.06 (0.18)	0	0.33
Exacerbations	0.44 (0.62)	0.13 (0.36)	0.24
Maintenance OCS dose (mg)	2.5 (5.18)	2.5 (5.18)	–
FEV_1_ (%), *n* = 7	78.8 (26.4)	67.6 (28.8)	*0*.*02*
ACQ score	1.6 (1.6)	1.5 (1.4)	0.43

With regards to asthma control as per ACQ score (ACQ < 1.5), 45.4% of benralizumab group were controlled pre and post switch compared with 50% of the mepolizumab group and 36.4% of the benralizumab and 37.5% of the mepolizumab group were uncontrolled despite switching. However, 18.2% of the benralizumab group improved their ACQ score at 6 months post switch with no one disimproving, while 1 patient who switched to mepolizumab disimproved with no one improving their ACQ score at 6 months post switch. In terms of exacerbations, 41.7% of those who switched to benralizumab did not have an exacerbation pre or post switch and 8.3% had an unchanged frequency. 62.5% in the mepolizumab group did not have an exacerbation pre or post switch with no change in frequency in 12.5%. 25% improved their exacerbation frequency following a switch from reslizumab to benralizumab and also 25% improved after switching to mepolizumab. There was no worsening of exacerbation frequency in the mepolizumab group but 25% of those in the benralizumab did have more frequent exacerbations after switching.

## Discussion

The results of this study show that the mean frequency of exacerbations, ACQ and FEV_1_ were unchanged six months post switching from reslizumab to mepolizumab or benralizumab. The dose of maintenance OCS increased due to one patient requiring an increased dose. In terms of improvements in ACQ and exacerbation frequency, while the ACQ remained unchanged for 84.2% of patients it did improve in 2 patients and the exacerbation frequency improved in 25%. When we analysed the groups as per individual biological agent, we found that the FEV_1_ reduced significantly in those who received mepolizumab by 12.2% despite 25% of this group reducing their exacerbation frequency post switch. The exact reason for this drop in FEV_1_ is unknown. However, it was noted that no patient improved their ACQ score post switching to mepolizumab but 12.5% did disimprove. As mepolizumab and benralizumab have a different mode of action and furthermore, factors which seem to predict poor outcomes in those treated with anti-IL-5 therapies seem to confer better outcomes in those treated with benralizumab, it is possible that underlying co-morbidities such as nasal symptoms may be a factor in this response (34).

Overall, our study is in line with other real-world studies where a small proportion of patients do not do well post switch but the vast majority maintain clinical benefit. One study of 60 patients switched from reslizumab or mepolizumab to benralizumab found that pulmonary function tests and asthma control improved, while OCS use reduced on switching (35). However, 16.67% did not respond following a switch to benralizumab from another agent (35). A further study found that in those switched to benralizumab, including three patients switched from reslizumab, there was no significant difference in lung function pre and post switch but there was a significant reduction in OCS use, exacerbations and 60% had significant improvement in ACT score (26). Most data on patients switching to mepolizumab involves a switch from omalizumab with an improvement in the clinical parameters seen in the majority of cases also (15, 28, 29).

Some patients respond very well to biological therapy, with some real-world studies stating more than 80% of patients will respond favourably while others respond partially or not at all (34, 36). Our study seems to be in line with existing data in this respect. For those whose symptoms did not improve following the switch in this study an alternative agent, and a further switch may be warranted. For our cohort, as the main indication for switching was to move from intravenous administration to subcutaneous administration, it was important that clinical effectiveness was maintained post switch. Although a few patients deteriorated post switch the vast majority remained clinically stable or improved.

This study is limited by small sample size of only 20 patients. Furthermore, there were a few missing data points which reduced the available data to 19 patients at some points. A further limitation is that we did not consider other co-morbidities such as body mass index and nasal symptoms in our analysis which are likely to have an impact on the effectiveness of a biologic agent in some patients.

However, there are a number of advantages to this study. This is real world data involving a switch from reslizumab to either benralizumab or mepolizumab. There is a lack of data regarding a switch from reslizumab in the literature and furthermore a switch to mepolizumab from an agent other than omalizumab. In addition, this cohort was a well-defined group who were followed up regularly.

In conclusion, switching from reslizumab to benralizumab or mepolizumab was clinically favourable in the majority of cases. Switching provided patients with a more convenient form of administration as reslizumab can only be given intravenously. Further and continued analysis would be warranted to assess response 12 months after switching.

## Data Availability

The original contributions presented in the study are included in the article further inquiries can be directed to the corresponding author/s.
